# The CAM cancer xenograft as a model for initial evaluation of MR labelled compounds

**DOI:** 10.1038/srep46690

**Published:** 2017-05-03

**Authors:** Zhi Zuo, Tatiana Syrovets, Yuzhou Wu, Susanne Hafner, Ina Vernikouskaya, Weina Liu, Genshan Ma, Tanja Weil, Thomas Simmet, Volker Rasche

**Affiliations:** 1 Department of Internal Medicine II, University Hospital Ulm, Ulm, Germany; 2 Department of Cardiology, Zhongda Hospital, Medical School of Southeast University, Nanjing, China; 3 Core Facility Small Animal MRI, Medical Faculty, Ulm University, Ulm, Germany; 4 Institute of Pharmacology of Natural Products and Clinical Pharmacology, Ulm University, Ulm, Germany; 5 Institute of Organic Chemistry III, Ulm University, Ulm, Germany

## Abstract

Non-invasive assessment of the biodistribution is of great importance during the development of new pharmaceutical compounds. In this contribution, the applicability of in ovo MRI for monitoring the biodistribution of MR contrast agent-labelled compounds was investigated in mamaria carcinomas xentotransplanted on the chorioallantoic membrane (CAM) exemplarily for Gd-DOTA and cHSA-PEO (2000)16-Gd after systemic injection of the compounds into a chorioallantoic capillary vein. MRI was performed directly prior and 30 min, 3 h, 5 h, 20 h, and 40 h after injection of the compound. The biodistribution of injected compounds could be assessed by MRI in different organs of the chicken embryo as well as in xenotransplanted tumors at all time points. A clearly prolonged enhancement of the tumor substrate could be shown for cHSA-PEO (2000)_16_-Gd. In conclusion, high-resolution in ovo MR imaging can be used for assessment of the *in vivo* biodistribution of labelled compounds, thus enabling efficient non-invasive initial testing.

Investigation of the bio-distribution of new pharmaceutical compounds is of paramount importance for e.g. assessment of the efficiency of specific substrate targeting. Labelling of new compounds with specific imaging markers, may enable rapid *in-vivo* assessment of its biodistribution after systemic administration.

A basic need for early investigating the properties of new contrast agents (CA) or labelled compounds is the availability of suited *in vivo* models. Using murine models is cumbersome especially considering the need for immune-deficient models in case specific uptake of CAs in e.g. human tumors is investigated. As an efficient alternative, chick embryonic models with xenotransplanted tumors on the chorioallantoic membrane (CAM) have raised interest over recent years as a substitute for murine cancer models. The model has been successfully applied to study cancer progression and its pharmacological tumor treatment^1^,^2^,^3^,^4^,^5^,^6^, angiogenesis^7^, or as a model system to study microsurgical instruments and techniques^8^. Compared to murine xenotransplantation tumor models, the CAM system offers various advantages. Most important, the full development of the lymphoid system does not complete before the late stage of incubation. Hence the chick embryo model is a naturally immune deficient host, enabling grafting of tissues without species-specific limitations and thus allowing xenotransplantation of many kinds of tumors^9^. In addition, the blood vessel network of the CAM provides an excellent environment for primary tumor formation and a basis for angiogenic blood vessel formation^10^. According to European law (Directive 2010/63/EU of the European Parliament and of the Council of 22 September 2010 on the protection of animals used for scientific purposes), the CAM model system represents an intermediate stage between isolated cultured cells and animals, which does not raise any ethical or legal concerns, thus being an attractive replacement for *in vivo* animal experiments. Considering the obvious advantages and the fact that the CAM model is comparatively cheap and easy to maintain, it may form an excellent platform for early investigation of the biodistribution of new contrast agents or labelled compounds.

Due its versatile image contrast and the increasingly improving amounts of available markers loadable to pharmaceutical compounds, magnetic resonance imaging (MRI) has gained increasing interest for non-invasive assessment and quantification of compounds after systemic administration. Gadolinium complexes are by far the most widely used contrast agents in clinical practice^11^. Because Gd^3+^ ions are toxic, they have to be linked into complexes with chelates (DTPA, DOTA, etc.) prior to application *in vivo*. Chelates of Gd^3+^ are currently used in nearly half of all diagnosis MRI procedures, especially in neoplastic disease^12^. The rather unspecific uptake of the current contrast agents do currently not allow specific enhancement of the target tissue. Ongoing research focuses on increasing the relaxivity of the CAs, while simultaneously aiming for functionalization of the compound to specific molecular targets thus ensuring higher concentration in the target substrate.

Despite its obvious advantages, the use of in ovo models for initial assessment of the biodistribution of labelled compounds by MRI after systemic injection has not been reported. Faucher *et al*.^13^ performed ultra-small Gd_2_O_3_ nanoparticle labelling of cancer cells *in vitro* with subsequent implantation of the labelled cancer cells onto the CAM before performing MRI. Kivrak-Pfiffner *et al*.^14^ reported intravascular injection of Gd-DOTA to evaluate the vascularization *in situ* after implantation of scaffolds. In their study, however, no analysis of the biodistribution of the contrast agent in the chick embryo was performed.

With the recent developments in immobilization of the chick embryo, non-invasive high-resolution MR imaging of the embryos and tumors planted on the CAM has rendered feasible^15^ and high-fidelity assessment of CA distributions after systemic injection can be approached.

In the presented work, the use of an in ovo tumor model for initial assessment of the biodistribution of MR labelled compounds after systemic injection was investigated. A commercial MR contrast agents (Gd-DOTA) and a self-assembled Gd-DOTA conjugated PbP (Protein based polypeptide copolymer) cHSA-PEO(2000)_16_-Gd were used to monitor the distribution of the compound over a period of 40 h after systemic injection. Gadolinium-metal chelates with 1,4,7,10-tetraazacyclododecane-N,N′,N′,N′′′-tetraacetic acid (Gd-DOTA) has been widely used for contrast-enhanced MRI for the detection of several malignant tumors, such as primary glioblastomas, intracranial metastases and hepatocellular carcinomas^16^,^17^. cHSA-PEO(2000)_16_-Gd is a newly developed nanoparticle introduced by Wu *et al*.^18^ which is formed by self-assembly of cHSA and PEO, loaded with Gd-DOTA^19^.

## Materials and Methods

All in ovo experiments were in compliance with the European directive for “Protection of animals used for experimental and scientific purposes” issued by the Directorate General for Environment (Directive 2010/63/EU of the European Parliament and of the Council of 22 September 2010 on the protection of animals used for scientific purposes. Official Journal L 276, 20.10.2010 p. 33–79 (revising Directive 86/609/EEC)) and the respective German interpretation “Tierschutz-Versuchstierverordnung vom 1. August 2013 (BGBl. I S. 3125, 3126), die durch Artikel 6 der Verordnung vom 12. Dezember 2013 (BGBl. I S. 4145) geändert worden ist”. Accordingly, in ovo experiments do not require any special additional allowance as long as the embryos are sacrificed before hatching as done in this study.

### Chick Embryos

Fertilized White Leghorn eggs (Gallus domesticus) were purchased from a hatchery (LSL Rhein-Main GmbH, Dieburg, Germany) and maintained at 37.8 °C and a 60% relative humidity atmosphere in a tabletop incubator for the whole incubation period. After incubation of the eggs for 4 days, they were gently cleaned with a 70% ethanol solution, fenestrated, and analyzed for fertilization and normal growth. Fertilization was checked by assessment of the CAM vascularization. After fenestration, the shell access windows were sealed with tape (“Leukopor”, BSN medical GmbH, Hamburg, Germany) to prevent contamination and placed back into the incubator. For the residual breeding time, the viability of the embryos was monitored daily by carefully checking the CAM vasculature for blood flow. The growth of the embryos was monitored according to Hamburger and Hamilton stages^20^ by candling the embryos and assessment of the shadows of internal structures in comparison with embryo growth standard.

### Cancer Cell Grafting

The human breast carcinoma cell line MDA-MB-231 (American Type Culture Collection, Rockville, MA) was cultured in L-15 growth medium supplemented with 10% (v/v) FBS. Grown cells were harvested using 0.25% trypsin/0.53 mM EDTA and washed at 37 °C in physiological saline solution before grafting.

On day 7 of incubation (d7), breast cancer cells we grafted onto the CAM. A silicone ring of 0.5 mm thickness with an inner diameter of 6 mm was placed on the chorioallantoic membrane of each egg. 2 × 10^6^ cells suspended in 20 μl of 50% Matrigel (BD Biosciences, Heidelberg, Germany) were seeded within the silicone ring. After cell grafting, the shell access windows were covered again and the eggs were kept in the incubator.

### Gd-DOTA conjugated micelles

cHSA-PEO (2000)_16_-Gd was prepared according to the protocol of Wu *et al*.^18^. HSA was cationized to obtain cHSA. cHSA was completely dissolved in degassed phosphate buffer (50 mM, pH 8.0), followed by adding MeO-PEO2000-NHS dissolved in DMSO. After reaction, cHSA-PEO (2000)_16_-GD was washed with deionized distilled water and then lyophilized to obtain cHSA-PEO (2000)_16_. Gd-DOTA was then conjugated onto cHSA-PEO (2000)_16_ via coupling to amino groups on the protein surface. Up to 85 DOTA-Gd could be incorporated into the complex. The resulting DOTA-Gd conjugated PbP (cHSA-PEO(2000)_16_-Gd) self-assembled into nano-sized core-shell structures in aqueous solution with the hydrophilic PEO shell outside and hydrophobic imaging core pointing to the inside from the protein backbone.

The longitudinal (r_1_) relaxivities of cHSA-PEO (2000)_16_-Gd samples were quantified at room temperature by a three-parameter fit of inversion recovery data of a dilution series containing Gd concentrations from 15 μM to 200 μM in 0.9% NaCl aqueous solution.

### Chick embryo intravascular injection protocol

In all investigations, chick embryos at d16 were used because at this time point the blood volume approaches the maximum during the incubation period^21^. After precooling, the shell access windows were enlarged without hurting the CAM to facilitate the injection of the CA. A Cold Light Source, KL 1500 LCD (SCHOTT, Mainz, Germany) was used for illumination. 30 G 1/2 needles (BD Microlance^TM^ 3, BD Drogheda, Ireland) and 0.01–1 ml injectors (B|BRAUN, Melsungen, Germany) were used for injection. CA was injected into a chorioallantoic capillary vein of medium size. After 5 minutes hemostasis by compression with cotton swab, about 200 μl OpSite* (Smith & Nephew, London, England) was applied to stop bleeding completely after injection. After ensuring that the bleeding was totally controlled, eggs were put back into the refrigerator.

### *In Ovo* MRI

Imaging was performed on an 11.7 Tesla small animal MRI system (Bruker BioSpec 117/16, Bruker Biospin, Ettlingen, Germany). Data were obtained with a 72 mm quadrature volume T/R resonator. GD^3+^ distribution was visualized applying a T_1_-weighted three-dimensional FLASH (3D-T_1_ FLASH) sequence with acquisition parameters as: TR/TE = 6/2 ms, matrix = 400 × 439 × 96, spatial resolution = 100 × 100 × 560 μm^3^ and NSA = 2. Prior to systemic injection of the compound high-resolution T_2_-weighted anatomic images were additionally acquired with a multislice rapid acquisition with relaxation enhancement (RARE) sequence. Scan parameters were as: TR/TE = 4320/45 ms, matrix size = 650 × 650, in-plane resolution = 77 × 91 μm^2^, slice thickness = 0.5 mm, no inter-slice gap, RARE factor = 8, and NSA = 4. For imaging, the eggs were placed into a custom-built polystyrene holder that was attached to the conventional animal support to ensure reproducible positioning. The eggs were cooled at 4 °C for 110 minutes before each MR investigation^15^ to prevent motion artifacts.

### GD^3+^ dose and timing optimization

Eight chick embryos were injected with different gadofosveset doses (5, 10, 20, 50, 60, 70, 80, and 90 μl of 0.5 mmol/ml gadofoveset solution) for assessment of the required contrast agent dosage. The required dosage was identified by analysis of the signal-to-noise ratio (SNR) in the umbilical vein.

Three further chick embryos were used for identification of the peak enhancement of the blood pool and the liver. Two embryos were scanned continuously from 10 to 180 minutes after injection, while scanning in the third embryo was performed continuously from 60 to 300 minutes after injection.

### Measuring the bio-distribution of systemically injected MR labelled compounds

Chick embryos at d16 with xenotransplanted breast carcinoma (cell line MDA-MB-231) were used for investigating the applicability of MRI for monitoring the bio-distribution of systemically injected MR-labelled compounds. In all chick embryos the described MR measurements were performed before (RARE, 3D-T_1_ FLASH) and at 30 minutes, 3 hours, 5 hours, 20 hours, and 40 hours after CA injection (3D-T_1_ FLASH).

The general applicability of the suggested approach was tested in ten chick embryos after gadofosveset (80 μl gadofesveset solution, 20 μMol Gd^3+^) injection. The application for investigation of new compounds was then evaluated in direct comparison of injecting Gd-DOTA (50 μl diluted solution, 0.18 μMol Gd^3+^) in ten and cHSA-PEO (2000)16-Gd (50 μl dilution, 0.18 μMol Gd^3+^) in eight chick embryos.

### Data analysis

**Figure 1:**
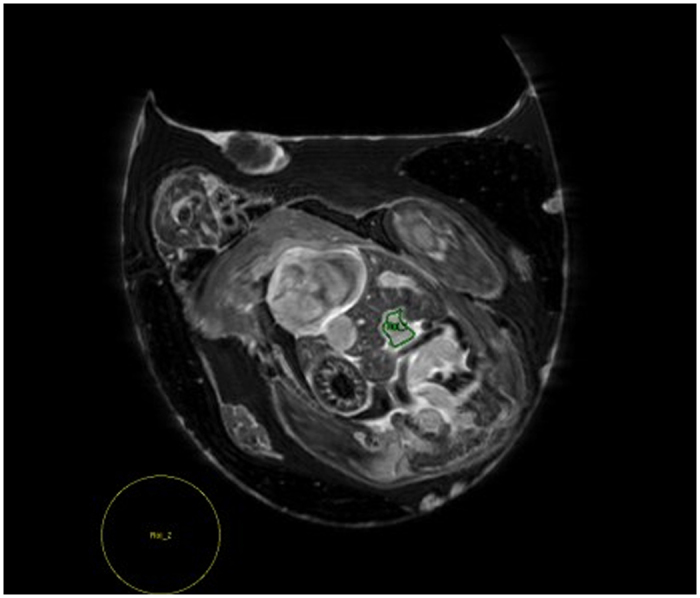
Example of choosing ROI (region of interest) of a vessel and of a background region. The ROI of the vessel was chosen covering most of the area in the biggest vessel in the liver (green), while the background was chosen outside of the egg in the area with the least artifact (yellow).

Signal-to-noise ratio (SNR) of different organs (vessels, allantoic fluid, liver, and brain) of the chick embryos at different time points after CA injection were determined. To calculate SNR, regions of interest (ROIs) were manually positioned by the same examiner at the same position at different time points. Special attention was paid to omit regions with artifacts. SNR was calculated according to: **SNR = (S**_**ROI**_ **- S**_**BG**_**)/δ**_**BG**_ with S_ROI_ and S_BG_ being the mean value over the ROI and background and δ_BG_ the standard deviation of the background. An example of choosing the respective ROIs exemplarily for the umbilical vein in the liver (green) and the background (yellow) is provided in Fig. 1.

In the breast cancer cell xenografts, the CA biodistribution at different time points was monitored. Time-SNR curves were generated for different embryo organs and the tumor (OriginPro 9.32, OriginLab, USA).

After finishing the 40 h scans, the solid tumors grafted on the CAM of the chick embryo were excised for histological workup at d17. Tissue sections were collected, paraffin embedded, and cut into 5 μm sections. Hematoxilin (nucleus) and eosin staining (cytoplasm), Ki-67 staining (proliferation), and desmin staining (angiogenesis) ^[4]^were obtained, analyzed microscopically by using an Axiophot microscope, and directly compared with the respective MR images.

Statistical significance of the differences of SNR was tested applying a unpaired two-tailed student’s t-test where p-values below 0.05 were considered statistically significant. All data are given as means +/− standard deviation (SD).

## Results

### Contrast agent dose assessment

**Figure 2:**
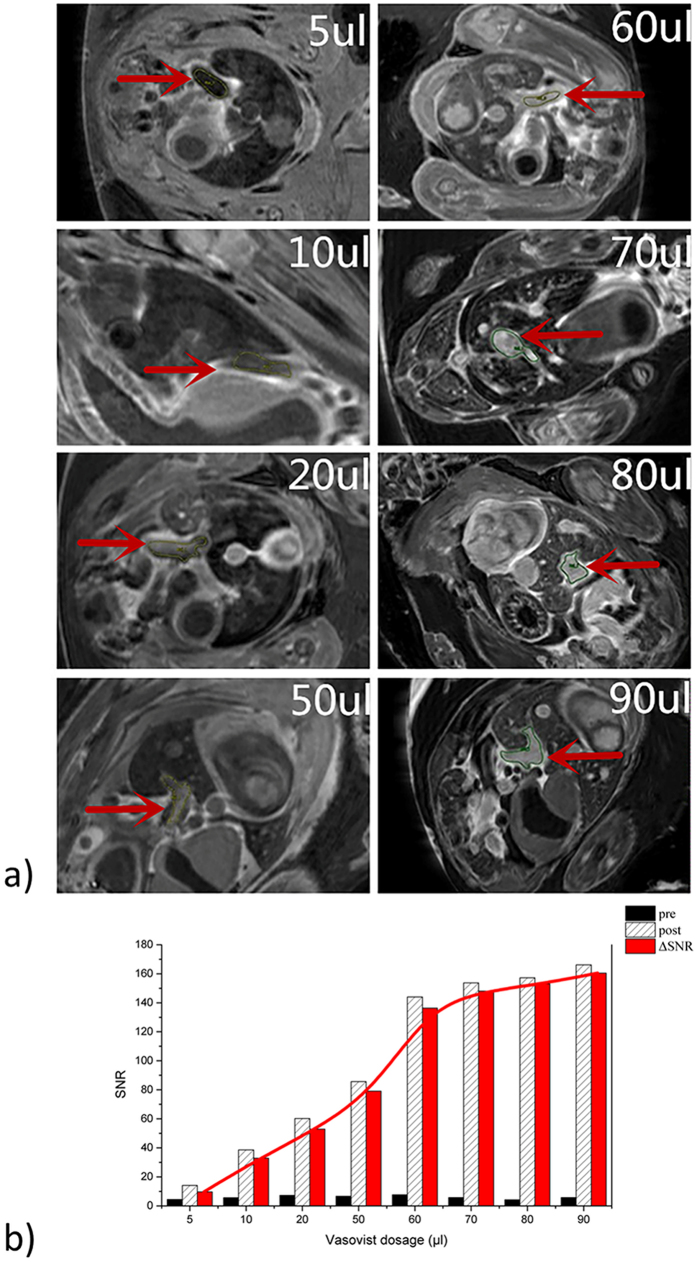
Images of chick embryos after systemic injection of gadofosveset, at different doses and the resulting SNR (Signal to Noise Ratio) of the umbilical veins. Blood pool enhancement increased with increasing dose till about 80 μl. Umbilical vein (red arrow) within the liver was chosen to calculate and compare relative SNR.

**Figure 3:**
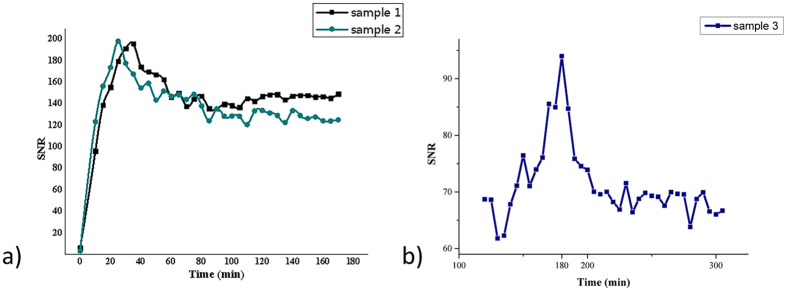
The SNR (Signal to Noise Ratio) - time curves of enhancement in blood and liver tissue. (**a**) shows SNR changes of the blood pool from injection to 3 h; (**b**) shows SNR changes of liver parenchyma from 120 to 305 min post-injection.

MR images of the embryos with different CA dose and resulting dose-SNR curves are provided in Fig. 2. The umbilical vein (red arrow in Fig. 2) was chosen for SNR assessment. SNR increased with increasing dosage approaching a plateau at 80 μl (20 μMol Gd^3+^), which was chosen as dose for subsequent experiments. The SNR-time curves in blood circulation and liver parenchyma are shown in Fig. 3. Maximal enhancement in the umbilical vein was observed about 30 minutes after injection Fig. 3a, whereas the liver parenchyma showed maximal enhancement 3 h after injection.

### Gadofosveset bio-distribution

**Figure 4:**
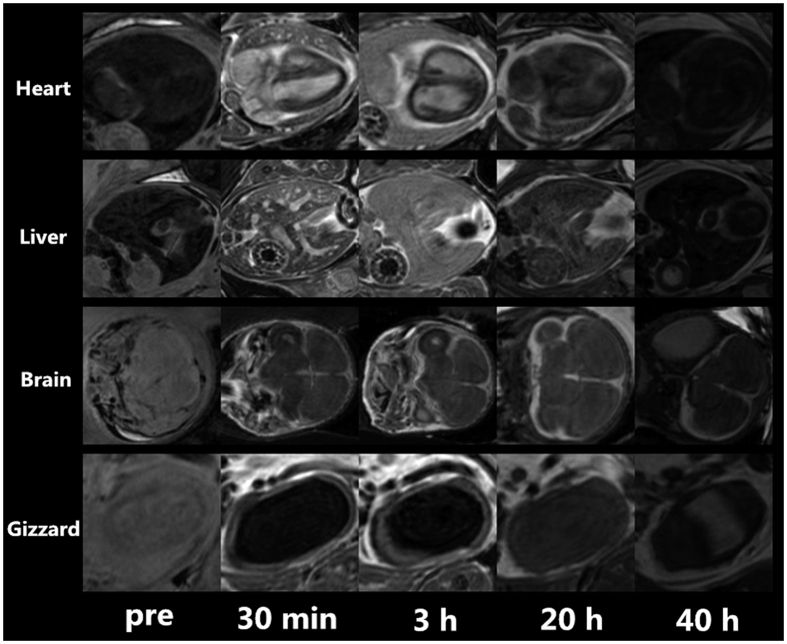
Gadofosveset biodistribution in different organs at different time points exemplarily shown for one chick embryo.

The CA biodistribution in different organs at different time points is exemplarily shown for one chick embryo in Fig. 4. The biodistribution of the injected high-dose (20 μmol Gd^3+^) gadofosveset can be clearly visualized with high spatial fidelity in ovo. From Fig. 4 an early enhancement of the blood circulation can be appreciated followed by enhancement of the liver parenchyma with final clearance into the allantoic fluid.

**Figure 5:**
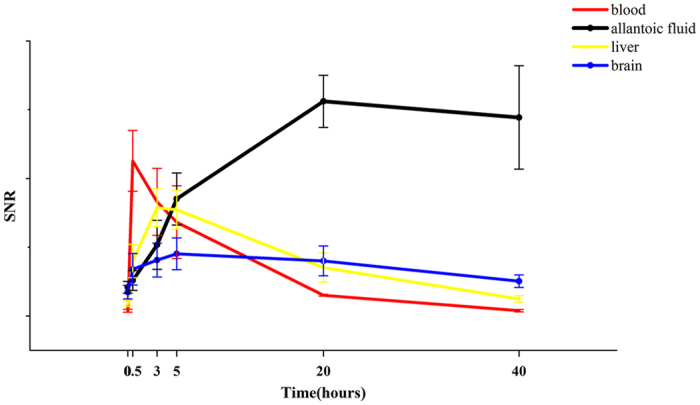
Time-SNR (Signal to Noise Ratio) curve for different organs from 0 to 40 h after systemic injection of gadofosveset (n = 10).

Change of the mean SNR ± stddev in different organs (vessels - red line, allantoic fluid - black line, liver parenchyma - yellow line, and brain - blue line) of the chick embryos at different time points after intravascular injection of gadofosveset is shown in Fig. 5.

**Figure 6:**
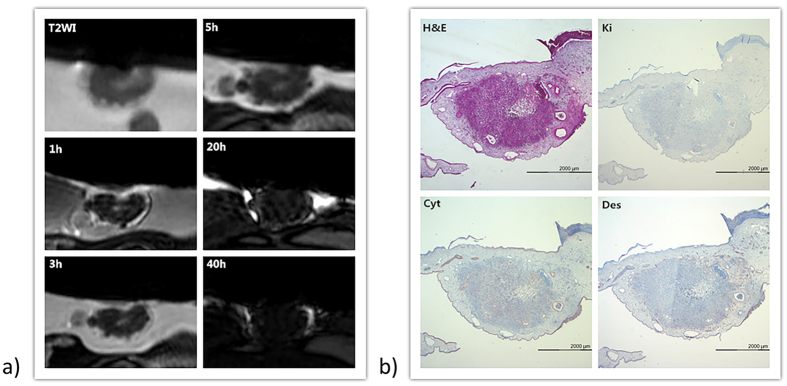
Correlation of immunohistological analysis and pre-CA (contrast agent) T_2_w image and post-CA T_1_w images of the tumor after systemic gadofosveset injection. White arrow: blood vessel; black arrow: loose cells area. (H&E: hematoxylin and eosin staining; Ki: Ki-67 staining; Cyt: cytokeratin staining; Des: desmin staining).

Images for the different investigated time points and immune-histological analyses of the tumor model are provided in Fig. 6. The dynamics of the enhancement of the tumor can be clearly appreciated. In direct comparison with the pre-administration situation, 30 minutes after systemic injection of the blood-pool contrast agent, the uptake in the tumor could be nicely visualized, and the enhancement gradually faded with increasing time. A clear enhancement of the border and central area of the solid tumor can be appreciated. The enhanced structures correlate well with the necrotic core and the CAM as identified in the immune-histological and T_2_ weighted images.

### cHSA-PEO (2000)_16_-Gd and Gd-DOTA

Molecular r_1_ relaxivity of the PbP at 11.7 T resulted as 5.93 mM^−1^s^−1^. Compared to the r_1_ relaxivity of clinically used Gd^3+^ contrast agents of Gd-DOTA (2.7 mM^−1^s^−1^)^22^ and Gadopentetate dimeglumine (4.5 mM^−1^s^−1^)^23^, the polymeric Gd^3+^ micelles showed increased relaxivity and hence MR efficiency. Compared to other gadolinium-based macromolecular contrast agents reported previously, the paramagnetic properties were at a comparable level.

**Figure 7:**
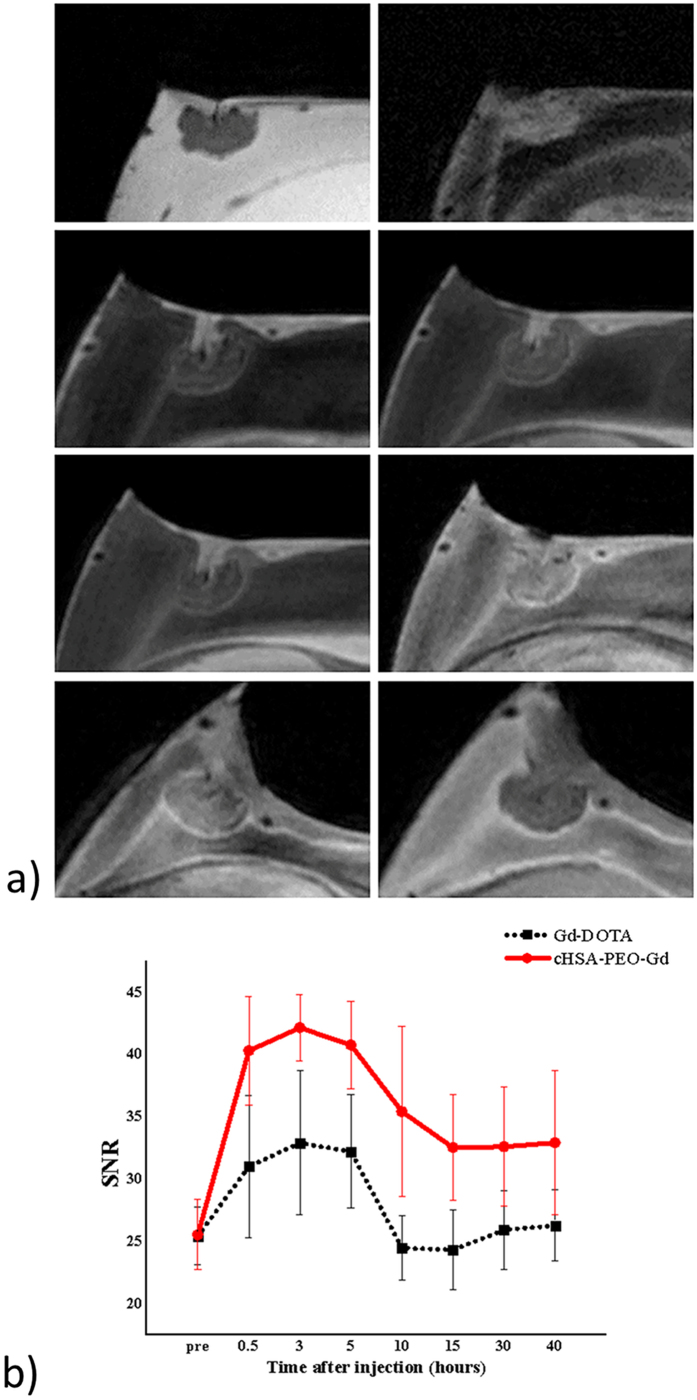
Representive MR images of cHSA-PEO(2000)_16_-Gd biodistribution in solid tumor tissuers (Left) and comparison of the SNR (Signal to Noise Ratio) of the tumor over the first 40 h after systemic injection of cHSA-PEO(2000)16-Gd and Gd-DOTA (Right).

A clear enhancement in the area of the xenografted tumor was observed after the injection of low-dose (0.18 μmol GD^3+^) cHSA-PEO (2000)_16_-Gd as well as Gd-DOTA. For both CAs a similar contrast enhancement with a peak tissue contrast at 3 h after injection was observed. However, where for cHSA-PEO (2000)_16_-Gd the enhancement persisted for at least 40 h post injection, the Gd-DOTA values returned to the pre injection values after 10 h. Detailed SNR data are provided in Table 1. Representative cHSA-PEO (2000)_16_-Gd enhanced tumor images and time-SNR curves are shown in Fig. 7. SNR values of the solid tumor tissues were significantly higher after injection for the cHSA-PEO (2000)_16_-Gd as compared to Gd-DOTA (p ≤ 0.01 for all contrast-enhanced time points).

## Discussion

With the rapid developments in targeted drugs, the assessment of the bio-distribution and pharmacokinetics of the compounds after systemic administrations is of utmost importance. With the increasing capabilities of linking MR labels to the compounds, there is a rising interest in using MRI for initial non-invasive monitoring of the fate of the injected drugs^24^. However, for assessing the drug properties, availability of e.g. appropriate tumor models is a prerequisite for testing diagnostic and therapeutic performance of new drugs. Currently, the subcutaneous transplant models in athymic nude mice as well as nude rats represent the gold standard for *in vivo* investigations in tumor research. Such models allow grafting of xenografts due to the marked immunodeficiency of the animals. However, according to the increasing demand to decrease or even prevent animal testing, the chick embryo system has been established as an alternative option for e.g. cancer xenograft research^4^. It provides rapid results and may be used as a simple inexpensive method practicable in any laboratory. Due to its obvious advantages it appears well suited to replace at least parts of the conventional *in vivo* studies. Until today the CAM system remains widely been used in tumor progression and metastatic research. Since the chick embryo is naturally immunodeficient, the CAM readily supports the engraftment of tumor tissues^25^. Most importantly, the CAM supports most cancer cell characteristics including growth, invasion, angiogenesis, and remodeling of the microenvironment. This makes the model exceptionally useful for investigating the molecular pathways of oncogenesis^26^,^27^,^28^,^29^. In recent years, particular emphasis has also been placed on tumor cell motility and its contribution to cancer metastasis^30^. Recent work in the laboratory of Harold Moses used the chick model to demonstrate that stromal cells can drive the outward migration of tumor cells^31^. All these research demonstrate the special position of CAM model in the research field of tumor.

However application of the CAM model for investigation of biodistributions of contrast agents is currently still underutilized. Kivrak-Pfiffner *et al*.^14^ reported a study involving intravascular injection of Gd contrast agent. They intravascular injected 100 μl Gd-DOTA to evaluate the vascularization *in situ* after implantation of scaffolds. They did not address the biodistribution of Gd-DOTA in the embryo. The work presented so far does not strongly support using the CAM model as a widely accepted *in vivo* model for novel MR contrast agent assessment.

With the novel developed immobilization protocol^15^, assessment of the biodistribution of MR CAs and labelled particles after systemic injection was investigated in this work. Intravascular injection techniques have long been used in the CAM system mainly for perfusion with fixatives^32^,^33^, assessment of anticancer medicines^3^, but, to our knowledge, never for the assessment of MR contrast agent biodistribution.

The optimal CA dosage suitable for chick embryos was investigated. Chick embryos at D16 were used for assessment because during that day the blood volume of chick embryo has developed to the mean peak value of 3.13 ml^21^ during the whole incubation period. Gadofosveset was chosen as representative Gd-based CA for estimation of the required doses. As a clinically used commercial blood-pool MR contrast agent, it has several advantages. It consists of a low molecular weight molecule chelated to Gadolinium that strongly binds with the plasma proteins, leading to a prolonged intravascular half-life yielding imaging windows after administration of about 30–60 min for patient application^34^,^35^. Compared to recommended *in vivo* dose of 0.12 ml/kg in humans, higher doses of at least 0.48 ml/kg were required to cause a pronounced enhancement in the chick embryo organs and the xenotransplanted tumors. Further increase of the dose till roughly 1 ml/kg appears reasonable if more subtle changes in CA aggregation should be visualized. The higher required dose is likely caused by the large distribution volume of the CA comprising the embryo as well as the surrounding fluids as well as the fact that embryonic blood vessels are known to be more leaky than mature ones^36^,^37^. Depending on the developmental stage, CAM vessels resemble undifferentiated, thin-walled capillaries with a monolayer endothelium incompletely surrounded by mesenchymal cells. Even though high CA doses were required for general application of CA, low-doses were sufficient for analysis of local CA aggregation e.g. in xenotransplanted tumors.

Maximal contrast enhancement was investigated for blood pool (30 min) and liver parenchyma (3 h) after systemic gadofosveset administration, indicating the need for careful planning of the acquisition times tailored to the target organ. Time-SNR curves for blood pool, liver parenchyma, brain, and allantoic fluid before and at 0.5, 3, 5, 20, 40 hours post-injection showing organ enhancement within the first 5 hours after injection. Slight enhancement was even observed in the brain likely due to the immature developed blood-brain barrier. After 20 hours, CA was gradually metabolized into the allantoic fluid.

In the cancer cell xenograft imaging, many details of tiny tumors were shown with different contrast. Gadofosveset accumulated mainly in the tumor surrounding tissue, which is closely attached to the CAM and which displays numerous blood vessels. Further enhancement was observed in the center part of solid tumor with low cell density.

The expected improved cell and tissue uptake of a Gd conjugated cHSA-PEO(2000)_16_-Gd could be clearly demonstrated in direct comparison with Gd-DOTA. As shown earlier for the poly-cationic serum albumin backbone material^37^, cHSA-PEO(2000)_16_-Gd showed higher uptake and retention time in the xenotransplanted tumors. Where the increased signal may partly be attributed to the higher relaxivity of cHSA-PEO(2000)_16_-Gd, the prolonged retention of at least 40 h clearly show the different pharmacokinetics, thus proving the applicability of MR imaging for initial in ovo assessment of the bio-distribution and pharmacokinetics of new compounds.

Thus, it could be shown that the introduced high-resolution MRI protocol enables in ovo assessment of the bio-distribution of MR-labeled compounds. The work proved the principal applicability of MRI for high-fidelity in ovo assessment of the bio-distribution of new compounds thus enabling efficient initial screening of compounds in the in ovo model.

## Additional Information

**How to cite this article:** Zuo, Z. *et al*. The CAM cancer xenograft as a model for initial evaluation of MR labelled compounds. *Sci. Rep.* **7**, 46690; doi: 10.1038/srep46690 (2017).

**Publisher's note:** Springer Nature remains neutral with regard to jurisdictional claims in published maps and institutional affiliations.

## Figures and Tables

**Table 1:** SNR (Signal to Noise Ratio) data of solid tumor tissues pre and at different time points after intravenous administration of the two different contrast agents.

Sample No.	pre	0.5 h	3 h	5 h	10 h	15 h	30 h	40 h
c1	24.43	26.78	29.52	24.65	24.60	21.25	25.60	28.43
c2	25.78	30.25	28.77	29.70	23.27	22.08	24.67	24.72
c3	22.04	27.20	30.02	33.43	23.51	21.49	26.21	22.58
c4	24.76	27.41	31.92	29.19	25.82	25.82	26.00	26.79
c5	27.76	31.93	33.63	30.88	26.06	26.42	28.16	25.31
c6	26.03	31.88	33.64	35.63	22.83	26.01	28.58	27.36
c7	28.34	45.90	48.36	41.76	24.94	21.65	19.59	25.66
c8	25.30	28.36	29.53	32.23	23.37	25.35	23.60	24.25
c9	28.04	32.99	33.76	34.19	29.99	31.05	31.51	32.83
c10	21.74	27.21	29.77	30.29	20.27	21.95	25.17	24.76
Mean (Gd-DOTA)	25.42	30.99	32.89	32.19	24.47	24.31	25.91	26.27
SD (Gd-DOTA)	2.30	5.72	5.77	4.55	2.56	3.18	3.18	2.84
e1	22.89	32.78	42.33	36.24	22.48	32.53	34.60	22.89
e2	26.07	37.12	40.25	41.93	38.26	26.66	26.01	26.07
e3	24.54	37.76	41.83	45.75	36.90	35.43	33.79	24.54
e4	25.92	42.23	41.09	40.69	32.60	32.62	31.37	25.92
e5	23.10	39.36	43.19	40.29	30.16	26.61	45.19	23.10
e6	24.03	44.15	45.06	40.63	37.22	33.22	33.69	24.03
e7	31.78	42.94	37.51	35.86	42.13	33.69	30.21	31.78
e8	25.74	45.96	45.81	44.59	43.49	39.43	34.59	28.47
Mean (PbPs)	25.51	40.29	42.13	40.75	35.41	32.52	32.58	32.92
SD (PbPs)	2.82	4.34	2.65	3.50	6.83	4.27	5.48	3.01
*p*	0.9	0.001	0.001	<0.001	0.006	0.001	0.006	0.01
